# Drought Stress Triggers Shifts in the Root Microbial Community and Alters Functional Categories in the Microbial Gene Pool

**DOI:** 10.3389/fmicb.2021.744897

**Published:** 2021-10-21

**Authors:** Jianbo Xie, Ghada E. Dawwam, Amira E. Sehim, Xian Li, Jiadong Wu, Sisi Chen, Deqiang Zhang

**Affiliations:** ^1^National Engineering Laboratory for Tree Breeding, College of Biological Sciences and Technology, Beijing Forestry University, Beijing, China; ^2^Key Laboratory of Genetics and Breeding in Forest Trees and Ornamental Plants, Ministry of Education, College of Biological Sciences and Technology, Beijing Forestry University, Beijing, China; ^3^Botany and Microbiology Department, Faculty of Science, Benha University, Benha, Egypt

**Keywords:** drought, microbiome, poplar, inoculation, interplay between drought stress and plant microbiome

## Abstract

Drought is a major threat to crop productivity and causes decreased plant growth, poor yields, and crop failure. Nevertheless, the frequency of droughts is expected to increase in the coming decades. The microbial communities associated with crop plants can influence how plants respond to various stresses; hence, microbiome manipulation is fast becoming an effective strategy for improving the stress tolerance of plants. The effect of drought stress on the root microbiome of perennial woody plants is currently poorly understood. Using *Populus* trees as a model ecosystem, we found that the diversity of the root microbial community decreased during drought treatment and that compositional shifts in microbes during drought stress were driven by the relative abundances of a large number of dominant phyla, including Actinobacteria, Firmicutes, and Proteobacteria. A subset of microbes, including *Streptomyces rochei*, *Bacillus arbutinivorans*, *B. endophyticus*, *B. megaterium*, *Aspergillus terreus*, *Penicillium raperi*, *Trichoderma ghanense*, *Gongronella butleri*, and *Rhizopus stolonifer*, was isolated from the drought-treated poplar rhizosphere soils, which have potentially beneficial to plant fitness. Further controlled inoculation experiments showed that the isolated bacterial and fungal isolates positively impacted plant growth and drought tolerance. Collectively, our results demonstrate the impact of drought on root microbiome structure and provide a novel example of manipulating root microbiomes to improve plant tolerance.

## Introduction

Drought stress is a serious and increasing problem in agriculture, as it negatively affects plant growth and development (Abid et al., 2019). Plant species are strongly associated with diverse archaeal, bacterial, and fungal communities (Veach et al., 2020). Microbiome communities are highly associated with plant health, productivity, and environmental adaptation (Berendsen et al., 2012) and have the potential to improve sustainable agricultural practices. Increasing evidence suggests that certain plant-microbe interactions can enhance plant stress tolerance, which has prompted researchers to manipulate microbial communities to promote plant growth during periods of environmental stress (Armada et al., 2018). The composition of plant-associated microbiomes is influenced by several host-associated and environmental factors (Baetz and Martinoia, 2013; Xu et al., 2018). Plants intimately interact with communities of microorganisms that share their environment (Naylor et al., 2017; Hu et al., 2018). Perturbations in plant metabolism, such as those triggered by abiotic stresses, can alter their microbiome with potential consequences for host fitness (Franzosa et al., 2019). Changes in soil moisture availability can significantly impact plant-associated microbiomes (Baetz and Martinoia, 2013; Santosmedellin et al., 2017; Xu et al., 2018). As drought stress represents a significant and increasing threat to crop productivity (Schwalm et al., 2017), current efforts are focused on improving the drought tolerance of various plant species (Rolli et al., 2015).

Plant species are highly associated with the microbial communities that inhabit rhizosphere (Berendsen et al., 2012; Cregger et al., 2018; Veach et al., 2019; Cregger et al., 2021). Variation in edaphic conditions selects for specific microbial communities. Increasing evidence demonstrates the importance of the microbiome, which consists of the entire complex of rhizosphere-associated microbes, their genetic elements and their interactions, in determining plant health (Berendsen et al., 2012). Several studies have shown that bacterial communities are dynamically shaped by several factors such as root exudates, soil, plant genotype, and chemotype (Veach et al., 2019). The complexity of the belowground plant microbiome enables its rapid adaption to changing soil conditions, which in turn can improve host adaptation (Timm et al., 2018; Veach et al., 2020). Therefore, environmental stressors are essential factors that could influence plant hosts or their microbiomes. Recent studies have demonstrated that drought has a strong impact on the structure of root microbiome communities and is associated with enrichment of specific bacterial lineages (Naylor et al., 2017; Santosmedellin et al., 2017; Edwards et al., 2018). Furthermore, plant root microbial ecosystems can act as “resource islands” for cultivating microorganisms in the surrounding soil (Marasco et al., 2012; Simmons et al., 2020). However, the structural and functional dynamics of microbial communities in perennial plant root systems during periods of abiotic stress are currently unknown. Alongside traditional breeding and genetic approaches, the manipulation of plant root microbiomes can potentially confer benefits capable of reducing the impact of abiotic stress on crops (Xu et al., 2018), thus enabling greater crop stability for feeding populations in drought-prone agroecosystems (Niu et al., 2017).

Poplars (*Populus* spp.) are model perennial systems for studying angiosperm tree physiology and genetics; they can also be employed to study the microbial composition of root rhizospheres and their responses to environmental changes (Munné-Bosch, 2007; Deyou et al., 2019; Wang et al., 2020a). Elucidation of these interactions is critical, as trees are economically essential to industry (Cregger et al., 2018). A variety of studies has examined total microbial communities associated with the roots of different species of *Populus* (Gottel et al., 2011; Armada et al., 2018; Veach et al., 2019, 2020; Tian et al., 2020). In nature, *Populus* species form mutualistic associations with specialized rhizosphere microbiomes that may affect nutrient uptake or water-absorbing efficiency from the soil under extreme conditions (Bissonnette et al., 2010; Timm et al., 2018; Wang et al., 2020b; Liu et al., 2021). Microbial community isolates from *Populus* have been shown to enhance the health, growth, and development of their plant hosts (Taghavi et al., 2009; Henning et al., 2016; Timm et al., 2016). Furthermore, the long-lived and clonal nature of *Populus* spp. could lead to functional relationships with specific microbes that may be underdeveloped or are absent in many annual model plant species that have typically served as functional organisms. Using *Populus* as a model system, this study seeks to characterize how the microbiome communities vary during drought stress treatment. We also studied the functional dynamics of the rhizosphere microbial communities using high-resolution metagenomic analyses. To determine whether some rhizosphere microbiomes contribute to changes in host phenotype under drought conditions, a series of functional bacterial and fungal strains were isolated and inoculated for host drought stress tests. We found that drought stress significantly shifted the composition of the *Populus* root microbiome. The drought-responsive microbes could potentially benefit their host as they may contribute to drought stress tolerance. These analyses revealed the dynamic interactions of microbial communities within the rhizosphere and changes in the functional gene pool under drought stress.

## Materials and Methods

### Soil Collection and Processing

Silt-clay loam soil was collected from 20 forest locations (at a depth of 30–75cm in 20 tree root balls), in Guan Xian County, Shandong Province, China (36°3'N, 115°47'E), using root cuttings from the entire natural distribution of *Populus tomentosa* (30–40°N, 105–125°E; Denyer et al., 2019). Then, the soils were sieved to <4mm, thoroughly homogenized and scooped into pots (24cm inner diameter; 28cm outer diameter; 26cm height). Soil pH was measured in a soil water (1:2.5 *w*/*v*) suspension using a pH meter. The elemental composition [concentrations of total nitrogen (N), phosphorus (P), and potassium (K)] of the soil was determined by a previously described method (Liu et al., 2021). Prior to seedling transplantation, sufficient water was added to submerge the soils. Physicochemical characteristics of the soil used for the growth of the plants were as follows: total N (mgkg^−1^), 842.5±9.3; available P (mgkg^−1^), 48.7±2.4; available K (mgkg^−1^), 354.7±2.4; and organic matter content (gkg^−1^), 9.2±9.1. The pH value was 0.75±0.06.

### Drought Treatment for *Populus tomentosa* Seedlings

#### Germ-Free Poplar Seedlings Preparation

The shoot tips collected from actively growing poplar were sterilized by washing in 10% bleach, 70% ethanol, and three times in deionized water. Tips were rooted in tissue culture medium (1× Murashige and Skoog basal salt mixture, 0.5mg/L indole-3-butyric acid) to produce rooted cuttings. Initial rooted cuttings were serially cultured in the same medium to generate germ-free experimental poplar seedlings. For this experiment, *P. tomentosa* rooted cuttings were transplanted into a growth chamber with sterilized soil for 7days, and healthy plants were selected for subsequent experiment.

#### Drought Treatment

The germ-free healthy plants were transplanted into individual pots with collected nature soils for 6months in a greenhouse at Beijing Forestry University, Beijing, China [16-h light/8-h dark; 20–24°C; 40°000'N, 116°200'E]. Nutrient water was supplied every 7days before drought treatment. Subsequently, soils were allowed to dry. Percent depletion of relative soil moisture contents (RSMCs) shown represents the proportion of potentially plant-available stored soil water in the ~5cm zones of the soil profile (the same zone in which root-associated microbiomes were sampled) estimated. Poplars were harvested at different time points: sample DA: RSMC 70–75% soil water content, 16/04/2019; sample DB: RSMC 50–55% soil water content, 27/04/2019; sample DC: RSMC 10–15% soil water content, 16/05/2019; and sample DD: rewatered for 2days (RSMC 30–35%), 06/06/2019. Poplars grown under standard watering conditions were included as a negative control and sampled at the same time points (CA; CB; CC; CD; RSMC 70–75%). Gravimetric water content of the soil was used to determine volumetric soil water percentage. Three biological replicates were used for each treatment.

### Sample Collection, Processing, and DNA Extraction

Plant samples were collected by manually extracting whole plants with their root systems. We collected rhizosphere samples (soil tightly adhering to the root surface) by collecting and pooling roots severed with sterile blades at each time point. Roots were vortexed in epiphyte removal buffer (0.75% KH_2_PO_4_, 0.95% K_2_HPO_4_, and 1% Triton X-100 in ddH_2_O; filter-sterilized at 0.2μM) for 5min and then centrifuged at 3,500rpm for 5min after removal of the root tissue to pellet the resulting rhizosphere soil. Bulk soil samples were collected ~5cm below the soil surface at distance from the sample plants. DNA extractions were performed immediately after sampling, following the DNeasy PowerSoil Kit (Qiagen, Hilden, Germany) protocol.

### Library Construction, Sequencing, and Shotgun Metagenomic Analyses

Aliquots of each DNA sample were mechanically sheared, and products were size-selected to 350bp and gel purified. Sequencing was performed using the Illumina HiSeq platform 2×150-bp sequencing technology. Quality control of raw reads was carried out as follows: removal of reads containing more than 40bp of low-quality nucleotides (sQ≤38); removal of reads containing more than 10bp 3' discarding 5'adapter and adapter-adapter contaminants. The qualified reads that passed the filters above were then assembled using MEGAHIT (Dinesh et al., 2017) with the parameter *--presets meta-large*. Clean data from each sample were realigned to scaftigs using Bowtie2^1^ to get unaligned paired-end reads with parameters *--end-to-end, --sensitive, -I 200*, and *-X 400*. All unaligned reads were used to assemble scaftigs. Gene prediction was conducted using MetaGeneMark,^2^ and gene redundancies were removed using CD-HIT^3^ with parameters *-c 0.95*, *-G 0*, *-aS 0.9*, *-g 1*, and *-d 0*. Gene catalogues with less than two read alignments were removed from the gene set. Species annotation was performed by searching the bacterial, fungal, archaeal, and viral sequences in the NR database using DIAMOND (Buchfink et al., 2015) with the parameter blastp *e*-value ≤1e^−5^. The assembled reads in our data set were 89.22% bacterial, 1.71% archaeal, 0.04% viral, and 8.9% unclassified.

### Amplicon Generation, Library Preparation, and Sequencing

Bulk soil DNA samples were amplified using a dual-indexed 16S rRNA Illumina iTags primer set specific to the V5–V7 region: 799F (5'-AACMGGATTAGATACCCKG-3') and 1193R (5'-ACGTCATCCCCACCTTCC-3'). For fungal libraries, the ITS1 region was amplified using universal primers. All polymerase chain reaction (PCR) reactions were carried out with 15μl of Phusion High-Fidelity PCR Master Mix (New England Biolabs), 0.2μM of forward and reverse primers, and ~10ng of template DNA. Thermal cycling consisted of initial denaturation at 98°C for 1min; followed by 30cycles of denaturation at 98°C for 10s, annealing at 50°C for 30s, and elongation at 72°C for 30s; and a final elongation at 72°C for 5min.

For PCR product quantification and qualification, the same volume of 1× loading buffer (containing SYBR green) was mixed with the PCR products and electrophoresed on a 2% agarose gel for detection. PCR products were mixed in equal ratios. The mixture of PCR products was purified with a Qiagen Gel Extraction Kit (Qiagen). For library preparation, sequencing libraries were generated using the TruSeq DNA PCR-Free Sample Preparation Kit (Illumina, San Diego, CA, United States) following the manufacturer’s recommendations. The library was sequenced on the Illumina NovaSeq platform, and 250-bp paired-end reads were generated.

### Sequence Processing for 16S rRNA

Paired-end reads were merged using FLASH (V1.2.7).^4^ Unjoined reads were discarded, and assembled reads were assigned to samples from barcodes by using split libraries. High-quality clean tags were subjected to the QIIME pipeline (V1.9.1).^5^ Tags were compared with the reference database (Silva database)^6^ using the UCHIME algorithm^7^ to obtain effective tags, and chimeric, mitochondria, and chloroplasts sequences were removed. Sequences with ≥97% similarity were assigned to the same operational taxonomic unit (OTU). Representative sequences for each OTU were screened for taxonomic annotation based on the Mothur algorithm. After taxonomies were assigned to each OUT, we discarded (1) all OTUs that were not assigned a Kingdom level RDP classification score of at least 0.5 and (2) all OTUs that were not assigned to Kingdom Bacteria. To remove low abundance OTUs that are in many cases artifacts generated through the sequencing process, we removed OTUs without at least five reads in at least three samples. These thresholds were found to be suitable using technical replicates in a data set published previously (Xu et al., 2018).

### Statistical Analyses

All analyses were conducted in the R Environment version 3.2.3. For principal coordinate analysis (PCoA), OTU or unigene counts were normalized using the variance-stabilizing transformation implemented in DESeq2. Weighted UniFrac distances were then calculated with phyloseq. Unconstrained PCoA was performed with the PCoA function in the *ape* package. Shannon diversity analysis was performed based on the *openxlsx*, *reshape2*, and *vegan* packages in R. Group difference analyses were performed using Metastats. The significance of different factors on community dissimilarity was tested with PERMANOVA or nested PERMANOVA using the “adonis” function of the VEGAN package in R based on weighted UniFrac distances.

### Isolation of Drought-Tolerant Microorganisms

The increased abundance of Actinobacteria and Firmicutes under drought conditions could be due to their high tolerance for stress, although direct selection from root exudates might have occurred. To investigate the potential contributions of drought-enriched microbes and their interactions with plants, we performed controlled inoculation experiments using seedlings grown in sterile soil. Bacterial and fungal species isolated from drought-stressed poplar roots were applied individually or in consortia to pre-sterilized soil. We collected rhizosphere samples (soil tightly adhering to the poplar root surface) of poplar seedlings under all drought treatment and pooling all root samples. Roots were washed with sterile water, ground in 10mM MgSO_4_ with a mortar and pestle and then centrifuged at 10,000×*g* for 10min to pellet the root-associated microbes. For bacterial isolation, 1ml of the suspension was plated onto King’s B medium and nutrient agar medium (24). For the isolation of fungal strains, 1ml of the suspension was plated onto potato dextrose agar (PDA) and malt extract agar (MEA) media supplemented with 50ppm of chloramphenicol to prevent bacterial growth and 35ppm of Rose Bengal to inhibit fast-growing fungi (Numponsak et al., 2018). Plates were then sealed with parafilm and incubated at 28°C for 7days. Representative colonies were picked and transferred to fresh nutrient agar medium for further studies.

### Evaluation of Water Stress in the Isolated Strains

To investigate the effects of water stress on the growth of the bacterial isolates, nutrient broth supplemented with varying concentrations of PEG-6000 (0, 15, 25, and 35%) was inoculated with 1% bacterial culture overnight. Cultures were incubated at 28°C with 120rpm of shaking. Growth was estimated by measuring the optical density (OD) of the cultures at 600nm using a spectrophotometer. The influence of water stress on fungal biomass was investigated by cultivating isolates in modified Melin-Norkrans medium supplemented with varying concentrations of PEG-6000 (0, 15, 25, and 35%). The cultures were incubated at 25°C in the dark for 7days. Mycelial mats were removed from the liquid medium, rinsed three times with distilled water, dried overnight at 80°C, and weighed (Zhang et al., 2011). The experiment was carried out using three repeats. Four bacterial and five fungal drought-tolerant strains were identified and selected for subsequent analyses.

### Genetic Characterization of Bacterial and Fungal Isolates

For bacterial strain identification, the 16S rRNA of the drought-tolerant bacterial strains was amplified *via* PCR using the following primers: 16S-F (forward, 5'-AGAGTTTGATCCTGGCTCAGAACGAACGCT-3') and 16S-R (reverse, 5'-TACGGCTACCTTGTTACGACTTCACCCC-3'). Each 20-μl PCR mixture contained 1μl of forward primer, 1μl of reverse primer, and 10μl of 2× Es Taq Master Mix. The PCR was programmed as follows: initial denaturation for 5min at 95°C, 36cycles of denaturation (0.5min at 95°C), annealing (0.5min at 55°C), and extension (1.5min at 72°C), and final extension for 5min at 72°C. PCR products were purified using a TaKaRa MiniBEST Agarose Gel DNA Extraction kit and analyzed *via* horizontal electrophoresis in 1% agarose gel. Similarities of the 16S rRNA sequences were examined using the National Center for Biotechnology Information BLAST program.

For fungal strain identification, genomic DNA was extracted using an EZgene TM Fungal DNA Miniprep kit according to the manufacturer’s instructions. Amplification of the fungal ITS region was performed *via* PCR using a forward primer (ITS1: 5'-TCCGTAGGTGAACCTGCGG-3') and a reverse primer (ITS4: 5'-TCCTCCGCTTATTGATATGC-3'; Wendrich et al., 2020). The thermocycling conditions were as follows: initial denaturation for 5min at 94°C, 35cycles of denaturation (1min at 94°C), annealing (40s at 55°C), and extension (1min at 72°C), and final extension for 5min at 72°C. The PCR products were analyzed using 1% agarose gel and sequenced using an ABI3730XL DNA Analyzer (Beijing Ribio Biotech). Similarities in the ITS sequences were examined using BLAST.

### Antagonism Tests for the Isolated Bacterial and Fungal Strains

Antagonism between selected fungal pairs was examined *in vitro via* the dual culture technique using PDA medium (Abdallah et al., 2015). A mycelial disc of one selected fungus (diameter 5mm) was deposited on one side of a Petri dish, and another fungal disc was deposited equidistantly on the other side of the same plate. After 7days of incubation at 28°C, antagonism was detected if zones of inhibited growth were present. For bacteria, strains were inoculated in lines on the surface of nutrient agar media. Other bacterial strains were inoculated in lines perpendicular to the initial inoculations. The plates were incubated for 24h at 37°C. Antagonistic activity was estimated by identifying zones of inhibited growth (Seyfferth et al., 2021).

### Measurement of Phosphate Solubilization and IAA Production

To measure the phosphate solubilization activity of the microbial isolates, both bacterial and fungal isolates were inoculated separately in Pikovskaya’s solid medium containing insoluble tri-calcium phosphate (0.5%). The solubilization zones and colony diameters were measured after 120h of incubation at 30°C. The results were expressed as solubilization efficiency (SE; Liu et al., 2020):


$$SE=\frac{\mathrm{Solubilization diameter}}{\mathrm{Growth diameter}}X\;100$$

Indole-3-acetic acid (IAA) is a primary plant hormone that regulates plant growth and development. To measure microbial IAA production, test strains were grown in MAZ medium (per liter: K_2_HPO_4_·3H_2_O, 6.0g; KH_2_PO_4_, 4.0g; MgSO_4_·7H_2_O, 0.2g; NaCl, 0.1g; CaCl_2_, 0.02g; FeCl_3_·6 H_2_O, 0.01g; H_3_BO_3_, 2.8mg; MnSO_4_·H_2_O, 2.1mg; NaMoO_4_·2 H_2_O, 2mg; ZnSO_4_·7H_2_O, 0.24mg; CuSO_4_·5H_2_O, 0.016mg) supplemented with 34mM malate (carbon source), 100μgml^−1^ tryptophan (IAA precursor), and 10mM NH_4_Cl (nitrogen source). Bacterial strains were inoculated into LB broth, while fungal strains were grown in Czapek’s medium amended with 1,000μgml^−1^ Trp (IAA precursor). The ability of isolates to produce IAA was determined using Salkowski’s reagent *via* a previously described colorimetric method (Rich-Griffin et al., 2020).

### Plant Soil Inoculation With Microbial Isolates

For the inoculation experiment, *P. tomentosa* LM50 tissue culture seedlings were transplanted into growth chamber with sterilized soil for 7days, and then, healthy seedlings were inoculated with bacterial and fungal strains. For bacterial inoculation, we inoculated 10ml of LB medium with a fresh colony and incubated the culture at 37°C overnight with 250rpm of shaking. The following day, 300μl of the culture medium was transferred to 10ml of LB medium and incubated for 6h, or until an OD (at 600nm) of 0.6 was reached. Then, 5ml of each culture was pelleted *via* centrifugation (2,000×*g*, 10min), and the pellet was resuspended in 1ml of sterilized water. For fungal inoculation, fungal isolates were cultured on PDA slants for 1week at 25°C. To prepare conidial suspensions for inoculation, approximately 9ml of sterile distilled water was added onto the surface of each fungal slant. The conidial suspensions were spread with a sterile glass rod and then filtered to remove mycelia. The spore suspension density was adjusted to 1×10^5^ml^−1^. The suspension was mixed with autoclaved water to a total volume of 400ml and added to the growth chamber. Plants were grown under a 16-h light/8-h dark photoperiod in a greenhouse at Beijing Forestry University, Beijing, China (40°00'N, 116°200'E). The suspension was added every 7days for the treatment group plants. After 30days, all plants except control samples were subjected to severe drought stress by suspending watering for 18days. Plants of the control group were watered with 400ml sterilized water every 7days.

### Plant Growth and Biochemical Parameters

Seven traits were measured after inoculation, and three biological replicates were used for each trait. Plant samples were collected by manually extracting whole plants with root systems. Plant height was measured from the apex to the base; root length was measured from the base to the longest root apex. Mature leaves from the same position were harvested and used to measure growth and physiological characteristics. The RWC (relative water content) of leaves was calculated using the following equation: RWC=(FW−DW)/(TW−DW)×100%; where FW=leaf fresh weight, TW=leaf turgid weight (leaves were placed in water for ~24h in darkness before weighing), and DW=leaf dry weight (leaves were dried for 24h at 90°C before weighing). Total protein concentration was measured by Bradford’s method (Gururani and Upadhyaya, 2013) using bovine serum albumin as the standard. The ascorbate peroxidase (APX), peroxidase (POD), and superoxide dismutase (SOD) activity levels were measured with customized kits (Nanjing Jiancheng Bioengineering Institute, Nanjing, China) according to the manufacturer’s instruction. Malondialdehyde (MDA) content was determined according to a previously described method (Beckers et al., 2017). The total sugar content was measured by anthrone colorimetry, as described previously (Munné-Bosch and Alegre, 2002). The proline content was measured by ninhydrin colorimetry using a previously described method (Dubrovina and Kiselev, 2016).

## Results

### Drought Stress Decreased Microbial Diversity Within the Rhizosphere

**Figure 1:**
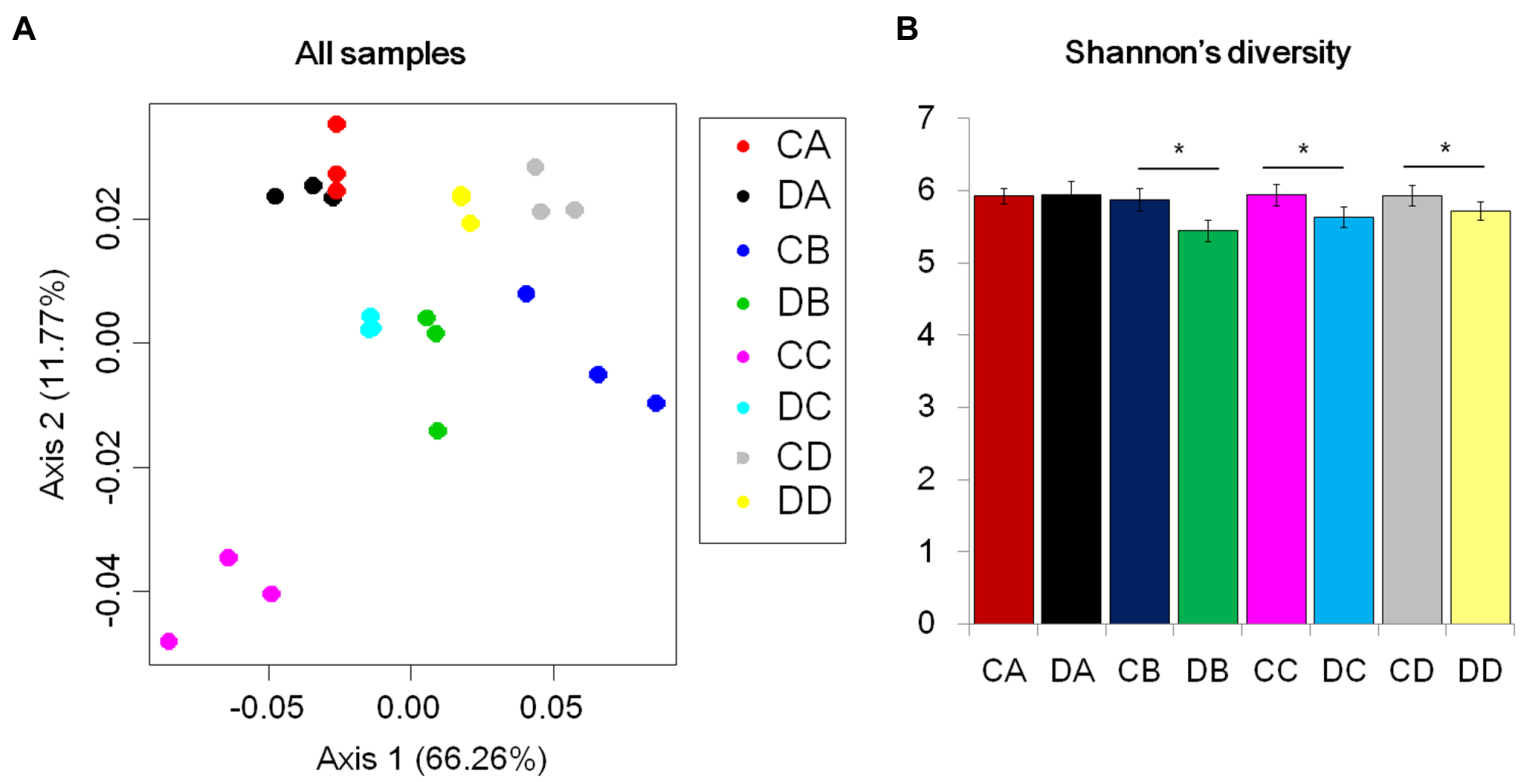
Drought impacts on root and soil microbiome development. **(A)** Principal coordinate analysis (PCoA) of Bray-Curtis distances for all control and drought samples. The color of each point indicates the control or drought treatment. **(B)** Mean Shannon’s diversity across the rhizosphere. (^*^p<0.05; ^*^Tukey’s test).

**Figure 2:**
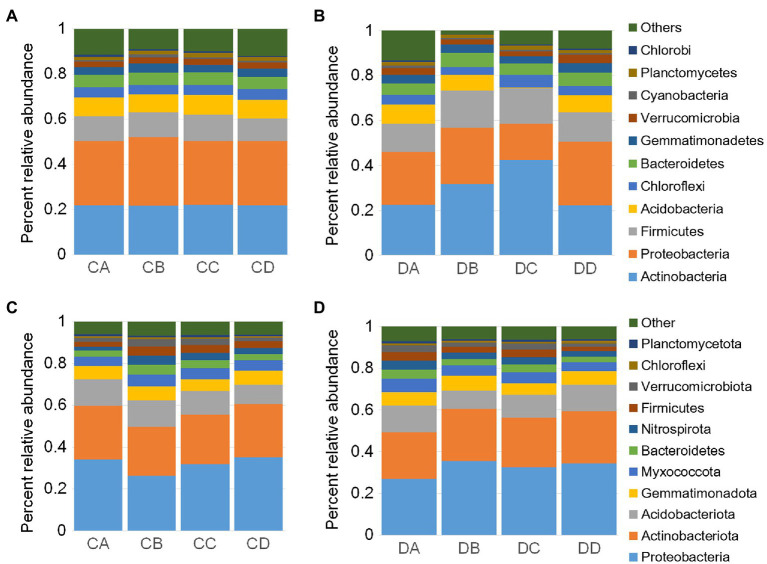
Drought impacts root microbiome development. Percent relative abundances of the top 11 most abundant phyla for rhizosphere samples of the control **(A)** and drought treatment **(B)**. Percent relative abundances of the top 11 most abundant phyla for bulk soil samples of the control **(C)** and drought treatment **(D)**. DA, drought sample A; DB, drought sample B; DC, drought sample C; DD, drought sample D; CA, control sample A; CB, control sample B; CC, control sample C; CD, control sample D.

To investigate the effects of drought stress on microbial recruitment in the rhizosphere, *Populus* seedlings were subjected to soil water deficiency at four RSMCs, selected according to previous research (Niu et al., 2017). Shotgun metagenomics was applied to examine community composition throughout the drought treatment. After quality control, a total of 155.98G 150-bp Illumina sequences were obtained (Supplementary Table S1). Assembly of the reads produced more than 642,520 scaffolds, ranging from 628.57 to 998.68bp in length (Supplementary Table S2). On average, 981,950 open reading frames were predicted (Supplementary Table S3). To explore the impact of RSMC on microbial community composition, unconstrained PCoAs were performed using Bray-Curtis distances. Samples taken from the drought treatments clustered into distinct groups according to treatment, and samples taken at the same timepoint approximately clustered into the same group. DA and CA samples were also grouped together (Figure 1A). This indicated that the microbial communities were strongly influenced by drought treatment and RSMC (Figure 1A). A comparison of drought and control treatments revealed that Shannon’s diversity level was significant difference between drought-treated samples and the control samples (Figure 1B; *p*<0.05, Tukey’s test). Shannon’s diversity level in the rhizosphere at the start was similar between treatment and control samples (Figure 1B); by contrast, levels of Shannon’s diversity in the soil remained largely unchanged (Supplementary Figure S1). We next examined the composition of the rhizosphere microbiome. At the phylum level, each community was dominated by Proteobacteria, Actinobacteria, Verrucomicrobia, and Firmicutes (Figures 2A,B). These bacterial phyla are relatively abundant and ubiquitous in soil (Figures 2C,D). Additional phyla, including Acidobacteria, Chloroflexi, and Bacteroidetes, were also found in nearly all samples, but their relative abundances were variable and typically represented less than 5% of the total reads (Supplementary Table S4).

### Drought Stress Induces Shifts in the Dominant Taxa

We then investigated temporal dynamics in the root microbiome under drought treatment by analyzing the relative abundance of each phylum in the microbial communities. The rhizosphere communities exhibited dynamic shifts during the drought treatment. Similar shifts were not observed for the control group, which indicated that this short period of plant development did not significantly influence the community composition (Figure 2A). To explore how bacterial community composition varied across both growth and drought treatment, NMDS ordinations and nested PERMANOVA analyses were performed (Supplementary Table S5). The analyses revealed significant differences in composition across the data set are driven primarily by drought treatment for rhizosphere (*F* statistic=6.216, *p*<0.001) and for bulk samples (*F* statistic=7.523, *p*<0.001). The compositional changes in the rhizosphere were largely due to dominant phyla such as Proteobacteria, Actinobacteria, Firmicutes, and Acidobacteria (Figure 2A). The compositional shifts in the microbial communities during the drought treatment were driven by the relative abundances of a large number of dominant phyla, including Actinobacteria, Firmicutes, and Proteobacteria (Figure 2A). At the class level, most of the bacteria belonged to Alphaproteobacteria, Betaproteobacteria, Gammaproteobacteria, Deltaproteobacteria, and Actinobacteria (Supplementary Figure S1A). Nitrosomonadaceae, Bacillaceae, Streptomycetaceae, and Xanthobacteraceae were the abundant families (Supplementary Figure S1B). Pseudolabrys, Streptomyces, Bacillus, and Gaiella were the abundant genera (Supplementary Figure S1C). Notably, we observed significant enrichment of Actinobacteria and Firmicutes, which was consistent with previous studies (Naylor et al., 2017; Santosmedellin et al., 2017; Cregger et al., 2018) and a likely contributing factor for the decrease in microbial diversity within the rhizosphere. Strikingly, after re-watering, the Actinobacteria and Firmicutes lineages in drought-treated plants decreased rapidly, by 12.3 and 10.6%, respectively (Figures 2A,B; *p*<0.01; one-sided Student’s *t* test).

At higher taxonomic resolution, we further identified specific bacteria that exhibit relative abundance patterns that differ between samples (Supplementary Data S1). Four genera were found enriched or depleted over the course of drought treatment, including *Bacillus*, *Actinotignum*, *Aneurinibacillus*, and *Streptomyces*. Strikingly, the most significantly enriched genera in drought treatment samples were *Streptomyces* and *Bacillus*, with 1.6- to10.4-fold higher abundance in drought-treated rhizosphere samples compared to control (Supplementary Data S1; *p*<0.001; Fisher’s exact test).

Samples of bulk soil were collected to determine the influence of drought on soil microbial communities, which were investigated using Illumina MiSeq sequencing of the V5–V7 region of the 16S rRNA gene. Proteobacteria, Actinobacteria, Acidobacteria, Gemmatimonadetes, Myxococcota, Bacteroidetes, Nitrospirae, Firmicutes, Verrucomicrobia, Chloroflexi, and Planctomyces were dominant phyla in soil samples (Figures 2C,D). Cyanobacteria were not dominant in soil samples (Figures 2C,D). We further observed that Proteobacteria was the most abundant phylum in soil, while Actinobacteria was most abundant in the rhizosphere (Figures 2A,B).

### Drought Treatment Induced Functional Shifts in the Rhizosphere Gene Pool

**Figure 3:**
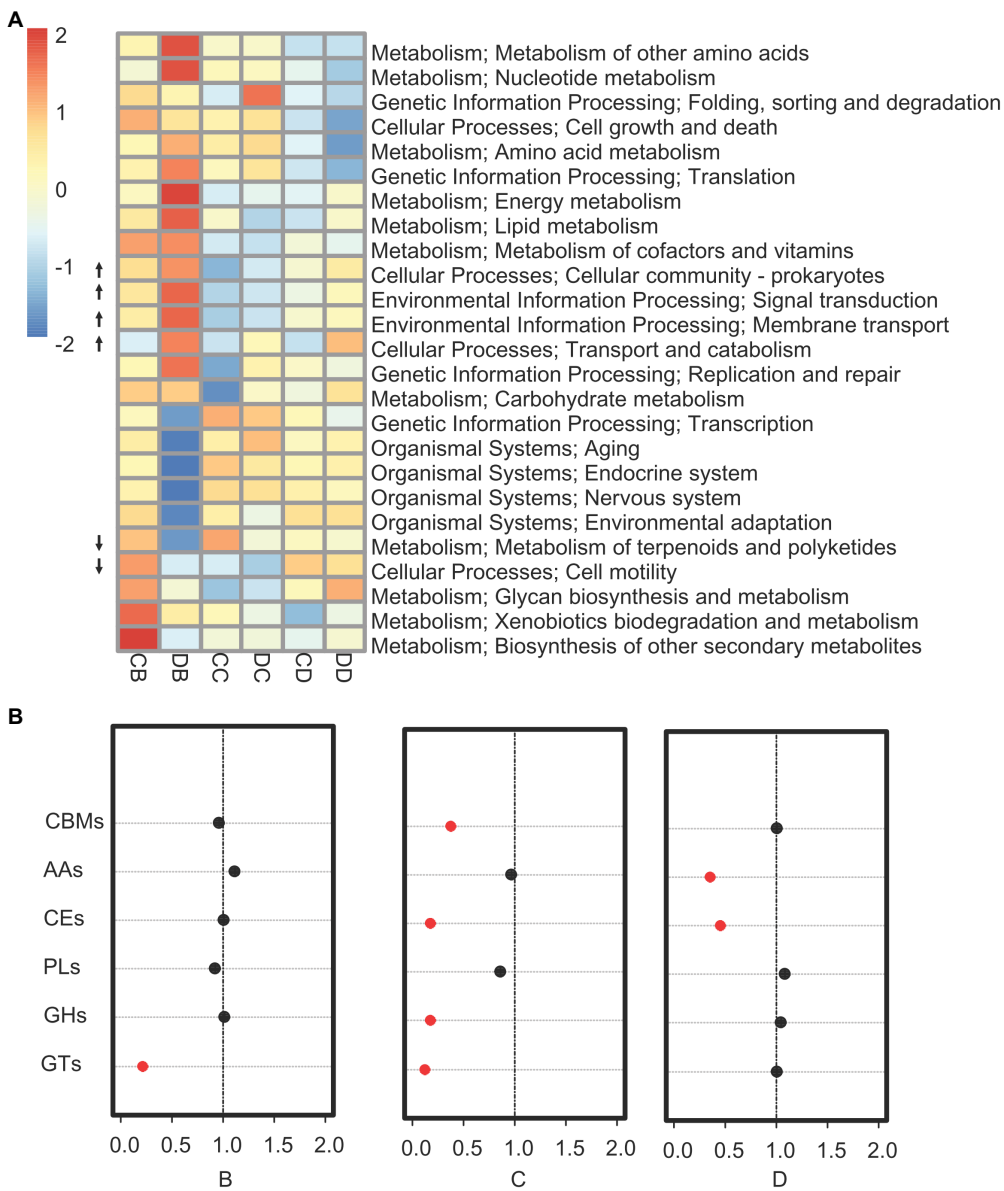
Drought impacts on the rhizosphere microbiome gene pool. **(A)** Kyoto Encyclopedia of Genes and Genomes enrichment analysis for all genes showing enrichment under drought for rhizosphere microbiomes. Categories significantly enriched are indicated (*p*≤0.05; Fisher’s exact test). The up (down) arrow indicates categories for which there are more (less) genes in the drought samples than in the control. **(B)** Enrichment analysis for all enzymes that degrade, modify, or create glycosidic bonds annotated in the CAZy database. The red circles indicate categories for which the enrichment had a value of *p*<0.05 in a Fisher’s exact test. The values on the *x*-axis indicate the fold enrichment ratio of annotated CAZy genes from samples B–D (drought treatment/control). CBMs, carbohydrate-binding modules; AAs, auxiliary activities; CEs, carbohydrate esterases; PLs, polysaccharide lyases; GHs, glycoside hydrolases; and GTs, glycosyltransferases.

To determine whether the drought-induced rhizosphere shifts correlated with changes in microbial function, we performed functional analyses on all samples. First, we investigated changes in rhizosphere community function during drought treatment. We found a significant increase in genes associated with signal transduction, membrane transport, and transport and catabolism. By contrast, pathways related to the metabolism of terpenoids and polyketides, as well as cell motility, exhibited a relative decrease in all three drought-treated samples (Figure 3A; *p*<0.01). The abundance of genes associated with the hydrolysis of carbohydrate esters, non-hydrolytic cleavage of glycosidic bonds, formation of glycosidic bonds, and hydrolysis and/or rearrangement of glycosidic bonds was significantly lower in drought-treated samples than in control samples (Fisher’s exact test: *p*<0.039; Figure 3B). A high-resolution EggNOG analysis of the functional categories influenced by drought in the rhizosphere revealed that the sub-functions of a large number of genes were related to resource transport, including phosphonate ABC transport, sulfate ABC transport, and putrescine ABC transport (Supplementary Data S2).

### Isolation of Drought-Tolerant Microorganisms From the Rhizosphere Under Drought Stress

The bacteria chosen include three strains from Firmicutes (*Bacillus megaterium* bc32, *Bacillus endophyticus* bc39, and *Bacillus arbutinivorans* bc49) and one Actinobacteria strain (*Streptomyces rochei* bc68); the fungal strains comprised *Aspergillus terreus* fg7, *Penicillium raperi* fg11, *Trichoderma ghanense* fg18, *Gongronella butleri* fg16, and *Rhizopus stolonifer* fg20 (Supplementary Figure S2; Supplementary Tables S6 and S7). No obvious antagonism was detected among the bacterial isolates (Supplementary Figure S3), whereas antagonism was present among all fungal isolates, with the exception of strains fg18 and fg20. Antagonism was also detected between some bacterial and fungal isolates. Thus, individual strains and representative groups of strains were chosen for this experiment.

To examine the potential growth-promoting effects of the selected microbial strains on plant development, levels of the plant growth hormone IAA were measured for each test strain using a colorimetric assay. IAA production was detected in all strains, with the highest levels detected in *B. endophyticus* bc39 (78.82μgml^−1^) and the lowest in *P. raperi* fg11 (13.42μgml^−1^). Six of the strains, *B. megaterium* bc32, *B. endophyticus* bc39, *B. arbutinivorans* bc49, *A. terreus* fg7, *P. raperi* fg11, and *T. ghanense* fg18, were able to solubilize phosphate. *Bacillus megaterium* bc32 and *A. terreus* fg7 were the most effective phosphorus solubilizers, exhibiting phosphorus solubilization indices of 165.57 and 150.36%, respectively (Supplementary Figure S4).

**Figure 4:**
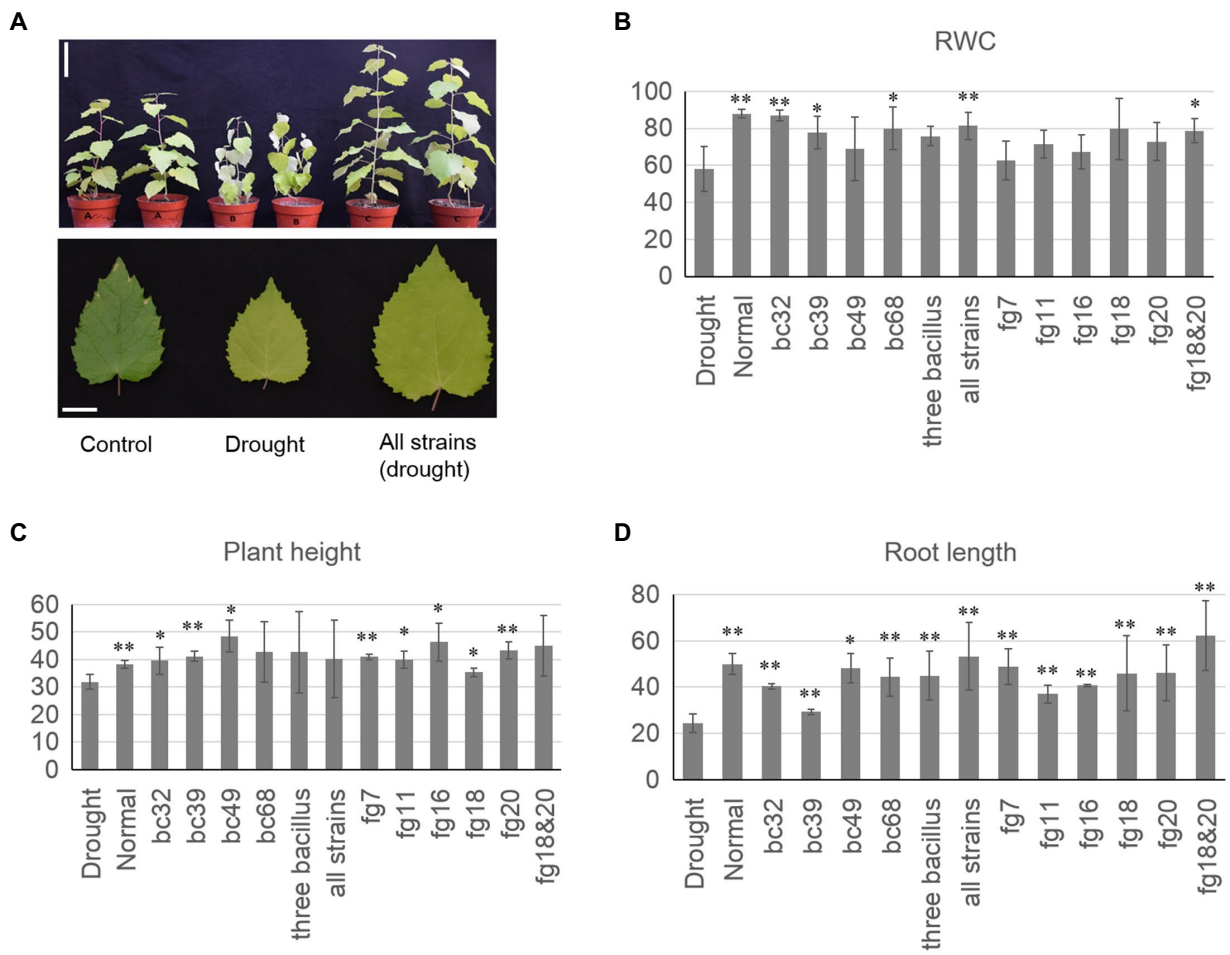
Isolated strains enhance the drought tolerance of poplar. **(A)** Inoculation of all mixed bacteria strains enhanced the drought tolerance of poplar. Bars (up)=10cm. Bars (down)=2cm. **(B)** The relative water content (RWC) of different inoculation groups. **(C)** The plant height of different inoculation groups. **(D)** The root length of different inoculation groups. RWC=[(fresh weight of leaves−dry weight of leaves)/(Turgid weight of leaves−dry weight of leaves)]×100. All strains (*Bacillus megaterium* bc32, *Bacillus endophyticus* bc39, *Bacillus arbutinivorans* bc49, and *Streptomyces rochei* bc68); three *Bacillus* (*Bacillus megaterium* bc32, *Bacillus endophyticus* bc39, and *Bacillus arbutinivorans* bc49; ^*^p<0.05; ^**^p<0.01).

Significant improvement in poplar growth parameters (plant height and root length) was observed following inoculation with several of the microbial species compared to the sterile control (Figure 4). We observed that the average root length of plants that were inoculated with all three bacterial strains simultaneously was 49.2% longer compared to the control (*p*<0.01; one-sided Student’s *t* test). Average root length was also 29.6% longer than the control in plants inoculated with consortia containing fg18 and fg20 (Figure 4; *p*<0.01; one-sided Student’s *t* test). Furthermore, six physiological characteristics, including leaf total proline, MDA content, APX activity, POD activity, sugar content, and SOD activity, were examined for each group (Supplementary Figure S4). All six characteristics differed significantly between inoculated plants and control plants under the drought treatment (*p*<0.05; one-sided Student’s *t* test). For example, the total proline content was significantly different in the inoculated group under drought treatment compared to the control drought treatment group (Supplementary Figure S5; *p*<0.05; one-sided Student’s *t* test).

## Discussion

In this work, we characterized the rhizosphere communities associated with a perennial plant under drought stress using high-throughput metagenomics. We found that the microbial community within the rhizosphere changed significantly during drought treatment, highlighting the dynamic nature of microbial communities associated with abiotic environments. Moreover, we isolated a series of bacterial and fungal strains from poplar roots that can directly or indirectly benefit plant growth. Our results suggest that the structure of the rhizosphere microbiome is relatively stable, while drought stress induces highly dynamic changes for some microbial lineages. In general, taxonomic classification represents the closest ancestor or relative in many cases. Despite the limitations, we identified similarities of rhizosphere microbial communities both in *Populus* and other genera, with strong preferential enrichment for Actinobacteria, Proteobacteria, Acidobacteria, and Chloroflexi (Zelicourt et al., 2013; Santosmedellin et al., 2017; Cregger et al., 2018; Xu et al., 2018). As a result, taxa belonging to these phyla were opportunistic during the drought stress. Firmicutes taxa were also comparatively abundant during the drought treatment. These taxonomic trends were observed across all soil types despite their intrinsic compositional differences, indicating that soil serves as a seed bank for rhizosphere recruitment. The result also indicates that the differences in the microbiome composition of the rhizosphere are not due to unwanted heterogeneity within the soil inoculum.

In our study, the drought treatment significantly influenced microbial community composition, which might have been due to the dramatic shifts in soil moisture. The coexistence of microbial species typically depends on metabolic trade-offs, where each species has fitness advantages under particular abiotic and biotic conditions (Xu et al., 2018). The enrichment of particular microbial strains may arise due to the differing life strategies of distinct lineages. For example, the spore-forming ability of Firmicutes and Actinobacteria enables them to enter a stable and quiescent state during periods of environmental stress; this strategy likely enabled them to persist under drought conditions, whereas less fit bacterial lineages decreased in abundance (Vanessa et al., 2013; Naylor et al., 2017). These bacterial communities likely carry a competitive advantage that allows them to respond more rapidly to environmental conditions (Wang et al., 2020b; Zhang et al., 2021). These may have the potential to confer benefits to a wide range of hosts as drought events become more prevalent globally (Lobell et al., 2011). Additionally, several members of Actinobacteria are characterized by their filamentous growth habit and their ability to produce stress-resistant spores (Hacquard et al., 2015; Santosmedellin et al., 2017), two traits that could help this group resist drought stress. Several other mechanisms, such as biofilm formation, and osmoprotectant production may be applied by the set of microorganisms enriched under drought conditions (Hacquard et al., 2015). The dynamic shifts in distinct lineages, including Actinobacteria and Firmicutes, could be host-specific and a result of differing drought-response mechanisms. Some microorganisms, such as Rhizophagus, Mesorhizobium, and Streptomyces, are reported to promote drought tolerance (Berendsen et al., 2012). One possible explanation of Actinobacteria and Firmicutes enriched under drought that is related to fast stress response of these lineages such as Bacillus. The two bacterial lineages composed almost entirely of monoderms, which have thick cell walls, and producing a large set of general stress proteins (Xu et al., 2018). Environmental changes shift the gene expressions of the plant host toward the production of cell wall components, plant hormones, and metabolic pathways of plant secondary metabolism (Satterlee et al., 2020; Shaw et al., 2021), which presumably has subsequent downstream effects on the plant-associated microbiome communities (Timm et al., 2018).

Metagenomics is powerful tool for understanding the functions of the rhizosphere (Nayfach and Pollard, 2015). In this study, we explored the functional diversity of root microbial communities during drought treatment. Most of the gene sequences were related to carbohydrate metabolism, including those encoding carbohydrate esterases, polysaccharide lyases, glycoside hydrolases, and glycosyltransferases. Carbohydrates, which participate in a multitude of biological processes, can combine to form an enormous number of compounds through the activity of carbohydrate-active enzymes (Lombard et al., 2014). Soil microbial communities play an important role in the carbon cycle, from serving as a carbon reserve to producing structural molecules (Lombard et al., 2014). Our results suggest that the drought treatment inhibited the gene pool associated with carbohydrate-active enzymes, implying that microbial metabolism and vital processes were less active in the drought-treated samples. Recent evidence has shown that environmental stress drives the changes in exudation of specific plant metabolites (Badri et al., 2013), further influencing the composition of the plant-associated microbiome. Pathways associated with transport and catabolism in the rhizosphere exhibited increased activity during the drought treatment, whereas signal transduction and metabolic pathways were reduced in drought-treated soil. Evidence showed that catabolic enzymes and catabolite transporters could be regulated by environmental changes (Maurer et al., 2005), which might contribute to tolerance to drought and other abiotic stresses in specific taxa. These functional groups might have been related to the stress responses of the microbial community in the drought-treated soil groups. Taken together, these data suggest that drought stress had a significant effect on the gene pool of rhizosphere-associated microbiomes and that rhizosphere genes associated with carbohydrate and amino acid metabolism and transport increased under drought.

Following the isolation of individual root microbial species, we investigated potential plant-microbiota interactions, as well as the effects of individual and multi-strain inoculation on plant growth during drought stress. We inoculated plants with four bacterial and five fungal strains that were abundant in the rhizosphere both individually and in combinations and then quantified the height, root length, and RWC of poplar seedlings. Following inoculation, the plants exhibited increased growth under drought treatment compared to the well-watered groups. It is possible that these strains promoted growth under drought stress through the production of specific plant hormones. Our analyses revealed that several strains were able to produce auxins and solubilize phosphate, two features that can enhance plant growth. The microbial production of other growth regulators, such as jasmonic and abscisic acids, can also improve plant performance under stressful soil conditions (Forchetti et al., 2010). In addition, extensive crosstalk exists among different signaling pathways (Selvakumar et al., 2014), leading to the possibility that specific signaling components may integrate inputs from multiple hormones to enhance plant tolerance. Several plant-promoting characteristics, such as ACC deaminase activity and siderophore-mediated iron assimilation, are widely distributed in bacteria and fungi. Examples include *Streptomyces* and *Amycolatopsis* species, which have been shown to enhance plant growth (Forchetti et al., 2010). Consistent with this, a recent study on the effect of drought on the microbiome observed a significant correlation between the relative abundance of the genus *Streptomyces* in plant roots and host drought tolerance (Xu et al., 2018). However, few studies exist in which bacteria and fungi were isolated from stressful environments and screened for their potential to produce ACC deaminase and siderophores. Our results suggest that inoculation of plant soil with specific strains or strain combinations can potentially benefit plant fitness, within the context of a controlled laboratory setting and given the absence of other microbes. Further investigation with a wide range of monoderm lineages will be necessary to understand the mechanisms driving microbe-associated drought tolerance.

## Data Availability Statement

The datasets presented in this study can be found in online repositories. The names of the repository/repositories and accession number(s) can be found in the article/Supplementary Material.

## Author Contributions

AS and GD performed the experiment. JX wrote the manuscript. DZ designed the experiment, obtained funding, and responsible for this article. XL and JW provided valuable suggestions to the manuscript. SC and DZ revised the manuscript. All authors contributed to the article and approved the submitted version.

## Funding

This work was supported by funding from the Project of the National Natural Science Foundation of China (Nos. 31872671, 31972954, and 32022057), Young Elite Scientists Sponsorship Program by CAST (2018QNRC001).

## Conflict of Interest

The authors declare that the research was conducted in the absence of any commercial or financial relationships that could be construed as a potential conflict of interest.

## Publisher’s Note

All claims expressed in this article are solely those of the authors and do not necessarily represent those of their affiliated organizations, or those of the publisher, the editors and the reviewers. Any product that may be evaluated in this article, or claim that may be made by its manufacturer, is not guaranteed or endorsed by the publisher.

## Footnotes

^1^ http://bowtie-bio.sourceforge.net/bowtie2/index.shtml

^2^ http://exon.gatech.edu/GeneMark/meta_gmhmmp.cgi

^3^ http://www.bioinformatics.org/cd-hit/

^4^ http://ccb.jhu.edu/software/FLASH

^5^ http://qiime.org/scripts/split_libraries_fastq.html

^6^ https://www.arb-silva.de

^7^ http://www.drive5.com/usearch/manual/uchime_algo.html
