# Supplementary material for: Drought Stress Triggers Shifts in the Root Microbial Community and Alters Functional Categories in the Microbial Gene Pool
Source: Front Microbiol. 2021 Oct 21;12:744897. doi: 10.3389/fmicb.2021.744897 (PMC8566882; doi:10.3389/fmicb.2021.744897)
Supplement: Supplementary file 1 [file Table_1.DOCX]

***Frontiers in Microbiology* Supporting Information**

Article title: Drought stress triggers shifts in the root microbial community and alters functional categories in the microbial gene pool

Jianbo Xie^1,2†^, Ghada E. Dawwam^1,2,3†^, Amira E. Sehim^1,2,3†^, Xian Li^1,2^, JiaDong Wu^1,2^, Sisi Chen^1,2^ and Deqiang Zhang^1,2*^

^1^National Engineering Laboratory for Tree Breeding, College of Biological Sciences and Technology, Beijing Forestry University, No. 35, Qinghua East Road, Beijing 100083, P. R. China;

^2^Key Laboratory of Genetics and Breeding in Forest Trees and Ornamental Plants, Ministry of Education, College of Biological Sciences and Technology, Beijing Forestry University, No. 35, Qinghua East Road, Beijing 100083, P. R. China.

^3^Botany and Microbiology Department, Faculty of Science, Benha University, Benha 13518, Egypt.

**The following Supporting Information is available for this article:**

**Supporting Figures:**

**Figure S1. Drought impacts on rhizosphere soil microbiome composition.** Percent relative abundance of the top 11 most abundant phyla for rhizosphere samples at the class (A), family (B), and genus levels (C). (D) Mean Shannon’s diversity across the bulk soil. DA: drought sample A, DB: drought sample B, DC: drought sample C, DD: drought sample D, CA: control sample A, CB: control sample B, CC: control sample C, CD: control sample D.

**Figure S2. Growth of isolated strains under drought stress.** The isolated bacteria (A) and fungi (B) were screened for drought tolerance using increasing concentrations of polyethylene glycol 6000 (PEG-6000; 0%, 15%, 25%, and 35%). Strains indicated with an asterisk (*) were selected for subsequent soil inoculation.

**Figure S3. Antagonism was detected among the bacterial isolates.** 32: *Bacillus megaterium*; 39: *Bacillus endophyticus*; 49: *Bacillus arbutinivorans*; 68: *Streptomyces rochei*; 50: *Bacillus megaterium.*

**Figure S4. Plant growth-promoting traits of the isolated strains.** (A) Quantification of indole-3-acetic acid (IAA) produced by bacterial strains. (B) Quantification of phosphorus solubilization efficiency.

**Figure S5. Plants inoculated with soil microbes exhibited increased tolerance to drought treatment.** Quantitative analysis of (A) leaf total proline, (B) malondialdehyde (MDA) content, (C) ascorbate peroxidase (APX) activity, (D) peroxidase (POD) activity, (E) sugar content, and (F) superoxide dismutase (SOD) activity (* *P* < 0.05; ** *P* < 0.01).

**Supporting Tables:**

**Table S1.** Quality filtered data produced by metagenome sequencing.

**Table S2**. The scaffolds assembly status.

**Table S3**. Predicted number of open reading frames for each scaffold.

**Table S4**. Proportion of sequences assigned to different microbial phyla.

**Table S5.** Effects of growth and drought treatment on the microbe community based on PERMANOVA.

**Table S6.** Optical density (OD) of cultures grown with different concentrations of polyethylene glycol 6000.

**Table S7.** The strains selected for soil inoculation.

**Supporting Data:**

**Data S1-S2** were provided in other formats (Excel), which were submitted as other separate files.

**Data S1**. Functional categories influenced by drought in the rhizosphere, as annotated using the EggNOG database.

**Data S2.** Differential abundance of bacterial taxa between groups.


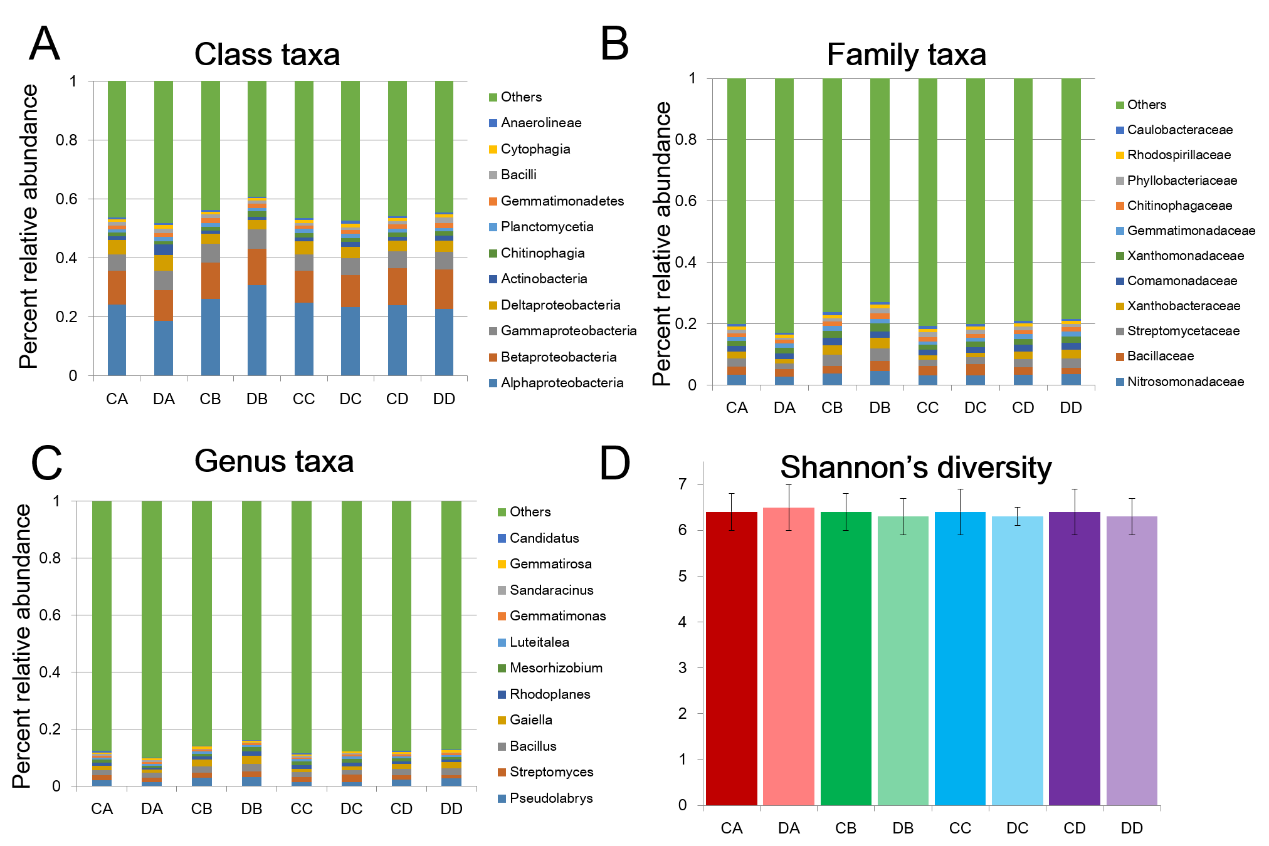


**Figure S1. Drought impacts on rhizosphere soil microbiome composition.** Percent relative abundance of the top 11 most abundant phyla for rhizosphere samples at the class (A), family (B), and genus levels (C). (D) Mean Shannon’s diversity across the bulk soil. DA: drought sample A, DB: drought sample B, DC: drought sample C, DD: drought sample D, CA: control sample A, CB: control sample B, CC: control sample C, CD: control sample D.


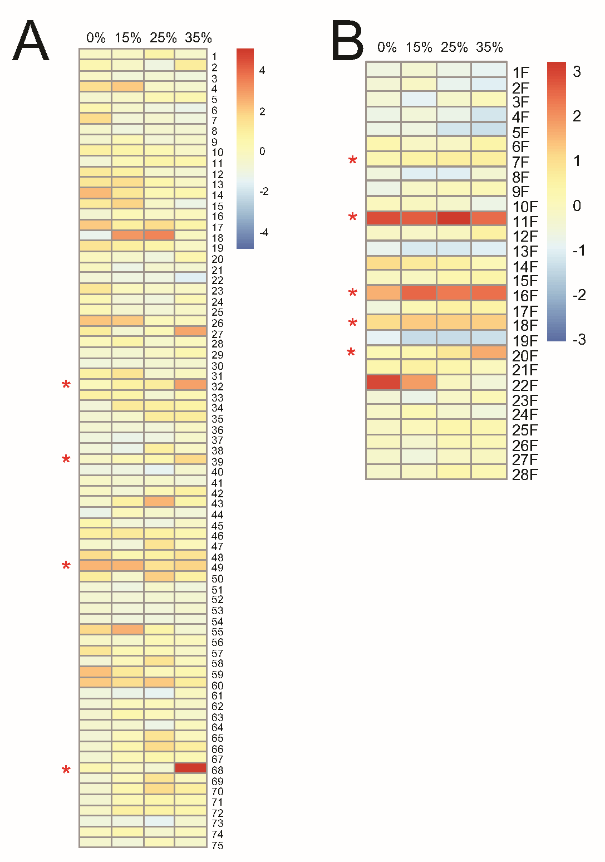


**Figure S2. Growth of isolated strains under drought stress.** The isolated bacteria (A) and fungi (B) were screened for drought tolerance using increasing concentrations of polyethylene glycol 6000 (PEG-6000; 0%, 15%, 25%, and 35%). Strains indicated with an asterisk (*) were selected for subsequent soil inoculation.


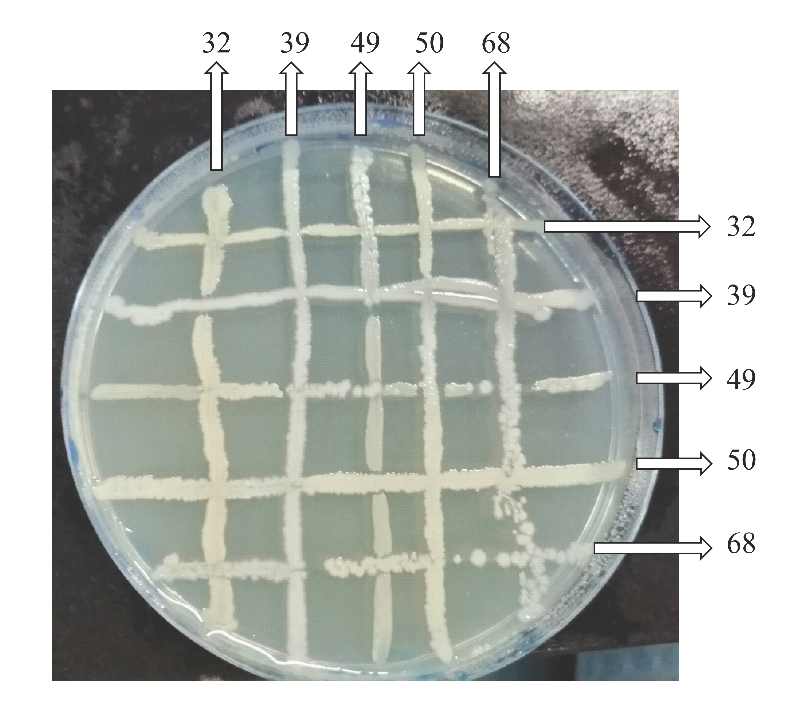


**Figure S3. Antagonism was detected among the bacterial isolates.** 32: Bacillus megaterium; 39: Bacillus endophyticus; 49: Bacillus arbutinivorans; 68: Streptomyces rochei; 50: *Bacillus megaterium.*


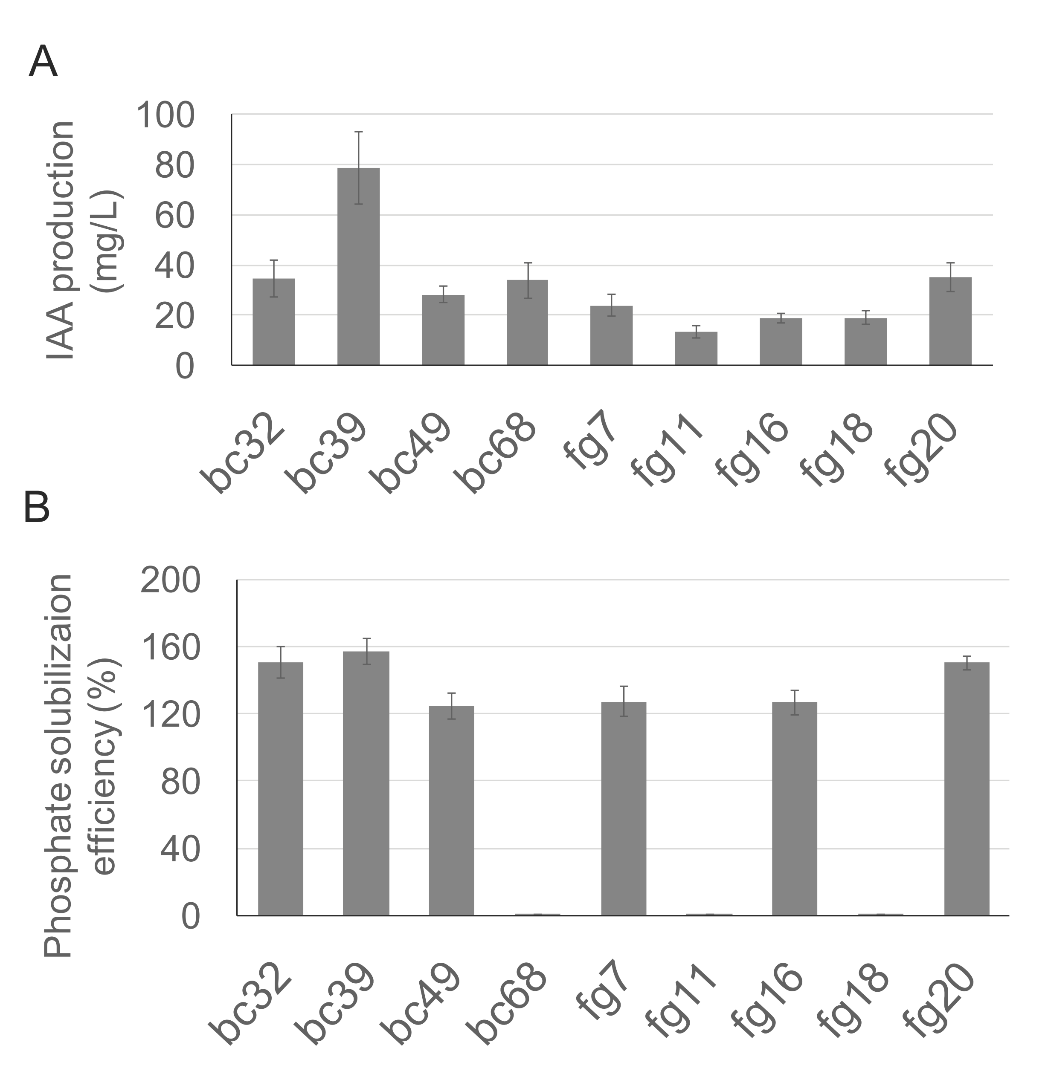


**Figure S4. Plant growth-promoting traits of the isolated strains.** (A) Quantification of indole-3-acetic acid (IAA) produced by bacterial strains. (B) Quantification of phosphorus solubilization efficiency.


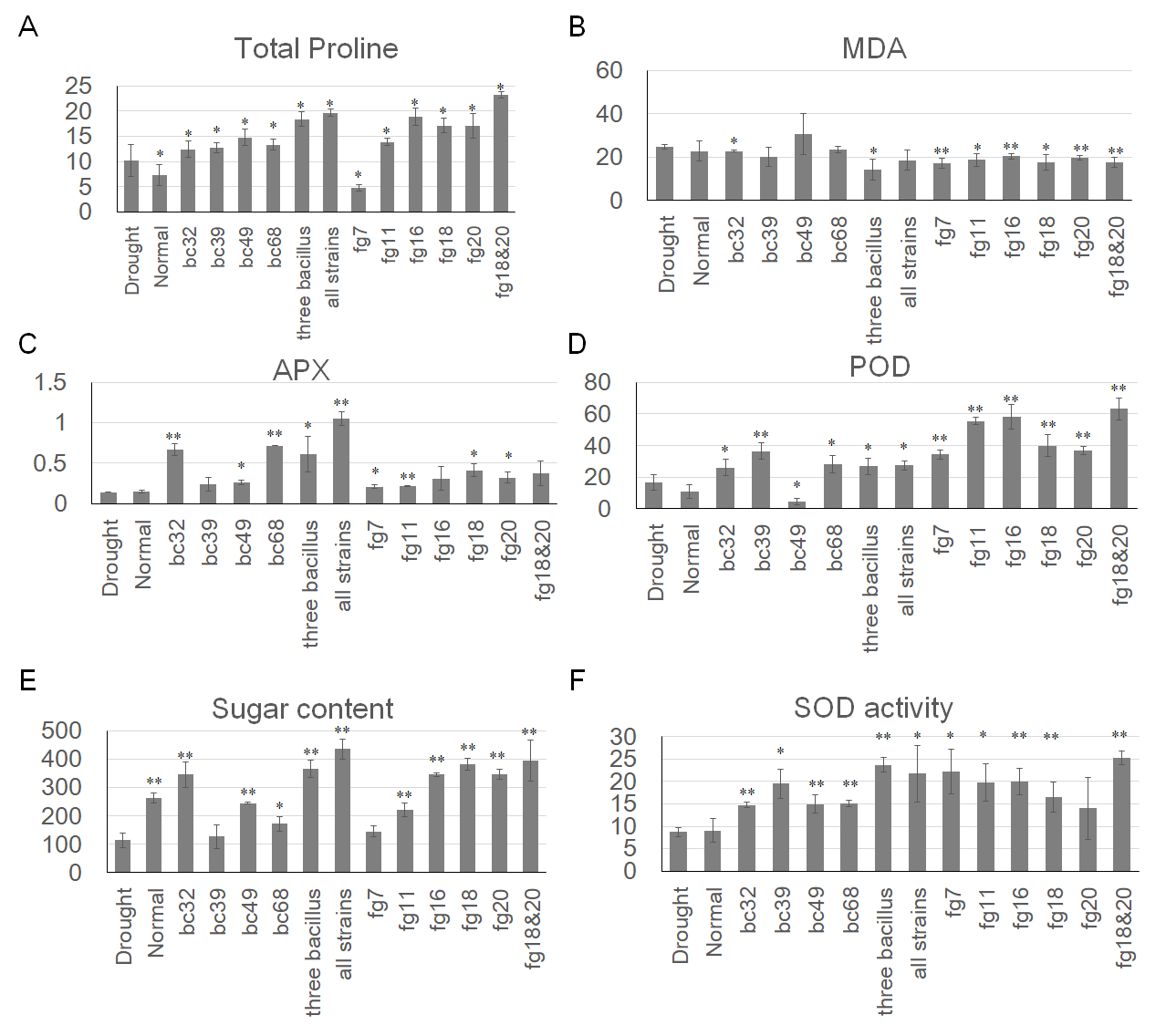


**Figure S5. Plants inoculated with soil microbes exhibited increased tolerance to drought treatment.** Quantitative analysis of (A) leaf total proline, (B) malondialdehyde (MDA) content, (C) ascorbate peroxidase (APX) activity, (D) peroxidase (POD) activity, (E) sugar content, and (F) superoxide dismutase (SOD) activity (* *P* < 0.05; ** *P* < 0.01).

Table S1. Quality filtered data produced by metagenome sequencing.

| **Sample** | **Raw Reads** | **Clean Reads** | **Error Rate(%)** | **Q30(%)** | **GC Content(%)** |
| --- | --- | --- | --- | --- | --- |
| CA1 | 40,314,704 | 40,238,313 | 0.03 | 92.32 | 42.97 |
| CA2 | 38,972,382 | 38,922,996 | 0.03 | 92.49 | 42.96 |
| CA3 | 37,172,084 | 37,099,101 | 0.03 | 92.79 | 43.17 |
| DA1 | 41,564,821 | 41,484,132 | 0.03 | 92.57 | 43.02 |
| DA2 | 38,309,880 | 38,248,254 | 0.03 | 92.34 | 42.97 |
| DA3 | 43,251,625 | 43,165,967 | 0.03 | 92.82 | 43.00 |
| CB1 | 63,999,722 | 63,973,444 | 0.03 | 93.74 | 61.38 |
| CB2 | 49,248,194 | 49,224,911 | 0.03 | 93.82 | 61.33 |
| CB3 | 59,827,031 | 59,803,750 | 0.03 | 94.28 | 61.40 |
| DB1 | 55,199,347 | 55,142,329 | 0.03 | 94.13 | 61.45 |
| DB2 | 72,244,385 | 72,210,248 | 0.03 | 94.00 | 61.40 |
| DB3 | 57,562,465 | 57,539,589 | 0.03 | 93.94 | 61.31 |
| CC1 | 54,361,826 | 54,329,110 | 0.03 | 93.75 | 60.70 |
| CC2 | 50,607,935 | 50,580,572 | 0.03 | 94.37 | 60.72 |
| CC3 | 60,904,725 | 60,877,224 | 0.03 | 93.06 | 60.42 |
| DC1 | 55,664,530 | 55,635,654 | 0.03 | 93.06 | 61.32 |
| DC2 | 44,135,421 | 44,114,875 | 0.03 | 94.03 | 61.39 |
| DC3 | 44,902,388 | 44,885,067 | 0.03 | 94.07 | 61.39 |
| CD1 | 52,297,504 | 52,277,542 | 0.03 | 94.19 | 61.41 |
| CD2 | 57,028,810 | 57,011,042 | 0.03 | 94.16 | 61.44 |
| CD3 | 52,194,632 | 52,178,671 | 0.03 | 93.77 | 61.41 |
| DD1 | 44,941,115 | 44,913,464 | 0.03 | 93.57 | 41.97 |
| DD2 | 46,795,517 | 46,694,255 | 0.03 | 92.61 | 43.17 |
| DD3 | 38,938,044 | 38,843,034 | 0.03 | 92.28 | 43.23 |

**Table S2**. The scaffolds assembly status.

| SampleID | Total len.(bp) | Number | Average len.(bp) | N50 Len.(bp) | N90 Len.(bp) | Max len.(bp) |
| --- | --- | --- | --- | --- | --- | --- |
| CC1 | 468,830,543 | 554,328 | 845.76 | 801 | 541 | 255,136 |
| CC2 | 416,103,263 | 502,118 | 828.70 | 784 | 540 | 743,156 |
| CC3 | 613,436,184 | 674,969 | 908.84 | 866 | 545 | 783,044 |
| DC4 | 749,728,588 | 838,045 | 894.62 | 853 | 547 | 569,822 |
| DC5 | 593,396,855 | 687,324 | 863.34 | 823 | 544 | 209,066 |
| DC6 | 595,191,312 | 649,637 | 916.19 | 878 | 549 | 293,796 |
| CB1 | 432,882,079 | 496,325 | 872.17 | 833 | 543 | 773,193 |
| CB2 | 417,948,838 | 470,836 | 887.67 | 846 | 544 | 290,737 |
| CB3 | 836,308,697 | 837,415 | 998.68 | 1,014 | 562 | 527,043 |
| DB1 | 619,617,263 | 710,214 | 872.44 | 843 | 547 | 573,859 |
| DB2 | 479,864,136 | 556,155 | 862.82 | 831 | 544 | 67,118 |
| DB3 | 578,863,334 | 646,254 | 877.82 | 839 | 562 | 78,134 |
| CD1 | 472,526,424 | 549,380 | 860.11 | 805 | 541 | 153,707 |
| CD2 | 535,366,415 | 602,178 | 889.05 | 841 | 545 | 562,843 |
| CD3 | 505,108,412 | 596,619 | 846.62 | 805 | 540 | 504,016 |
| DD4 | 542,275,971 | 622,581 | 871.01 | 837 | 544 | 96,219 |
| DD5 | 497,556,599 | 582,454 | 854.24 | 802 | 541 | 436,151 |
| DD6 | 629,889,808 | 726,305 | 867.25 | 823 | 541 | 362,056 |
| CA1 | 671,263,854 | 745,474 | 900.45 | 869 | 548 | 451,922 |
| CA2 | 846,238,473 | 880,721 | 960.85 | 942 | 554 | 361,472 |
| CA3 | 675,194,628 | 709,013 | 952.3 | 933 | 554 | 159,825 |
| DA1 | 710,537,147 | 761,608 | 932.94 | 922 | 553 | 442,652 |
| DA2 | 525,480,358 | 626,211 | 839.14 | 803 | 542 | 501,006 |
| DA3 | 729,114,991 | 758,097 | 961.77 | 953 | 556 | 845,885 |
| NOVO_MIX | 175,207,826 | 278,742 | 628.57 | 606 | 517 | 31,613 |

**Table S3**. Predicted number of open reading frames for each scaffold.

| Sample name | ORFs Number | Total length | Average length | GC |
| --- | --- | --- | --- | --- |
| CA1 | 1,136,997 | 597.66 | 525.65 | 61.57 |
| CA2 | 1,396,171 | 759.28 | 543.83 | 62.15 |
| CA3 | 1,120,635 | 604.47 | 539.4 | 61.85 |
| DA1 | 1,197,826 | 636.73 | 531.57 | 61.03 |
| DA2 | 934,839 | 469.69 | 502.43 | 61.29 |
| DA3 | 1,209,718 | 653.08 | 539.86 | 61.97 |
| CB1 | 751,598 | 386.37 | 514.06 | 60.7 |
| CB2 | 714,947 | 371.74 | 519.95 | 60.08 |
| CB3 | 1,361,803 | 750.18 | 550.87 | 62.7 |
| DB1 | 1,078,627 | 553.93 | 513.55 | 61.97 |
| DB2 | 845,032 | 429.68 | 508.48 | 60.97 |
| DB3 | 863,142 | 433.69 | 502.45 | 61.88 |
| CC1 | 827,520 | 419.57 | 507.02 | 61.17 |
| CC2 | 740,036 | 373.69 | 504.97 | 61.95 |
| CC3 | 1,040,107 | 549.71 | 528.52 | 61.62 |
| DC1 | 1,280,557 | 675.42 | 527.45 | 63.3 |
| DC2 | 1,025,629 | 533.78 | 520.44 | 63.1 |
| DC3 | 1,002,102 | 536.23 | 535.1 | 63.45 |
| DD1 | 820,426 | 424.58 | 517.52 | 61.27 |
| DD2 | 924,278 | 481.87 | 521.35 | 61.87 |
| DD3 | 891,382 | 453.93 | 509.24 | 61.73 |
| CD1 | 940,154 | 487.34 | 518.37 | 62.95 |
| CD2 | 873,400 | 448.69 | 513.72 | 62.66 |
| CD3 | 1,094,308 | 566.44 | 517.62 | 62.84 |
| NOVO_MIX | 358,698 | 156.11 | 435.21 | 63.15 |

| **Table S4**. Proportion of sequences assigned to different microbial phyla. | | | | |
| --- | --- | --- | --- | --- |
| Phylum | CA | CB | CD | CD |
| Actinobacteria | 0.218982 | 0.215516 | 0.220554 | 0.219293 |
| Proteobacteria | 0.284363 | 0.304535 | 0.281436 | 0.283411 |
| Firmicutes | 0.1078637 | 0.109417 | 0.116147 | 0.100025 |
| Acidobacteria | 0.083683 | 0.079486 | 0.089413 | 0.080921 |
| Chloroflexi | 0.045326 | 0.04055 | 0.042601 | 0.048486 |
| Bacteroidetes | 0.055276 | 0.054292 | 0.05748 | 0.05345 |
| Gemmatimonadetes | 0.034676 | 0.041554 | 0.030424 | 0.037784 |
| Verrucomicrobia | 0.023541 | 0.028144 | 0.027471 | 0.027036 |
| Cyanobacteria | 0.006874 | 0.014217 | 0.008318 | 0.007314 |
| Planctomycetes | 0.01554 | 0.015806 | 0.020314 | 0.018036 |
| Chlorobi | 0.007775 | 0.005285 | 0.006625 | 0.004451 |
| Others | 0.1161002 | 0.091198 | 0.099217 | 0.119793 |
|  | DA | DB | DC | DD |
| Actinobacteria | 0.225149 | 0.316869 | 0.425344 | 0.222983 |
| Proteobacteria | 0.234666 | 0.25164 | 0.161307 | 0.282686 |
| Firmicutes | 0.126126654 | 0.164646 | 0.156547 | 0.131266 |
| Acidobacteria | 0.084211 | 0.070875 | 0.003086 | 0.074713 |
| Chloroflexi | 0.044575 | 0.033174 | 0.055965 | 0.042138 |
| Bacteroidetes | 0.049753 | 0.063312 | 0.050548 | 0.058717 |
| Gemmatimonadetes | 0.03783 | 0.037795 | 0.033545 | 0.043976 |
| Verrucomicrobia | 0.031365 | 0.022244 | 0.021506 | 0.035929 |
| Cyanobacteria | 0.010702 | 0.006468 | 0.007595 | 0.006088 |
| Planctomycetes | 0.01667 | 0.013333 | 0.017608 | 0.013842 |
| Chlorobi | 0.006815 | 0.002991 | 0.005614 | 0.00629 |
| Others | 0.132137346 | 0.016653 | 0.061335 | 0.081372 |

**Table S5**. Effects of growth and drought treatment on the microbe community based on PERMANOVA.

| Variables | Rhizosphere soil samples | | | Bulk soil samples | | |
| --- | --- | --- | --- | --- | --- | --- |
|  | F value | R^2^ | Pr (>F) | F value | R^2^ | Pr (>F) |
| Growth | 1.221 | 0.0289 | 0.154 | 1.256 | 0.032 | 0.176 |
| Drought treatment | 6.216 | 0.456 | <0.001 | 7.523 | 0.524 | <0.001 |
| Growth×Drought treatment | 1.819 | 0.039 | 0.096 | 1.792 | 0.037 | 0.112 |

The significance of growth and drought treatment effects on bacterial community dissimilarity was tested with PERMANOVA or nested PERMANOVA (based on weighted UniFrac distances).

**Table S6**. Optical density (OD) of cultures grown with different concentrations of polyethylene glycol 6000.

| Bacterial strain | 0 | 0.15 | 0.25 | 0.35 |
| --- | --- | --- | --- | --- |
| 1 | 0.2176 | 0.1667 | 0.1589 | 0.0829 |
| 2 | 0.299 | 0.1562 | 0.0911 | 0.1297 |
| 3 | 0.2154 | 0.1178 | 0.1077 | 0.0657 |
| 4 | 0.4592 | 0.3316 | 0.1199 | 0.0786 |
| 5 | 0.1648 | 0.1566 | 0.1462 | 0.1077 |
| 6 | 0.3022 | 0.1466 | 0.0944 | 0.0552 |
| 7 | 0.4724 | 0.1287 | 0.1127 | 0.0645 |
| 8 | 0.1864 | 0.115 | 0.112 | 0.0765 |
| 9 | 0.1745 | 0.1928 | 0.103 | 0.0831 |
| 10 | 0.3704 | 0.2276 | 0.1453 | 0.0855 |
| 11 | 0.1364 | 0.1958 | 0.1602 | 0.1157 |
| 12 | 0.4058 | 0.24 | 0.1156 | 0.0745 |
| 13 | 0.4214 | 0.2969 | 0.155 | 0.0893 |
| 14 | 0.5702 | 0.2742 | 0.1437 | 0.111 |
| 15 | 0.4043 | 0.3197 | 0.1186 | 0.0598 |
| 16 | 0.1494 | 0.195 | 0.1251 | 0.0962 |
| 17 | 0.526 | 0.2542 | 0.2008 | 0.1167 |
| 18 | 0.0559 | 0.4015 | 0.272 | 0.0925 |
| 19 | 0.4345 | 0.2496 | 0.1602 | 0.0891 |
| 20 | 0.2388 | 0.1459 | 0.0959 | 0.1102 |
| 21 | 0.2316 | 0.0963 | 0.111 | 0.0747 |
| 22 | 0.1393 | 0.1033 | 0.0909 | 0.0441 |
| 23 | 0.4183 | 0.1752 | 0.1214 | 0.0879 |
| 24 | 0.2788 | 0.1282 | 0.0969 | 0.102 |
| 25 | 0.2729 | 0.1421 | 0.106 | 0.0865 |
| 26 | 0.5513 | 0.3294 | 0.1354 | 0.0953 |
| 27 | 0.3917 | 0.1318 | 0.1409 | 0.1864 |
| 28 | 0.2575 | 0.2313 | 0.1167 | 0.0951 |
| 29 | 0.1646 | 0.1303 | 0.1265 | 0.1085 |
| 30 | 0.1177 | 0.1192 | 0.1094 | 0.0791 |
| 31 | 0.3629 | 0.2791 | 0.1114 | 0.0912 |
| 32 | 0.2464 | 0.2449 | 0.1771 | 0.1902 |
| 33 | 0.1058 | 0.2218 | 0.1075 | 0.1557 |
| 34 | 0.3616 | 0.2256 | 0.1081 | 0.1056 |
| 35 | 0.1275 | 0.2624 | 0.1726 | 0.1215 |
| 36 | 0.1511 | 0.1459 | 0.1785 | 0.1313 |
| 37 | 0.1547 | 0.1279 | 0.1006 | 0.0872 |
| 38 | 0.1196 | 0.0869 | 0.0921 | 0.0822 |
| 39 | 0.1972 | 0.1901 | 0.1546 | 0.1534 |
| 40 | 0.1263 | 0.0929 | 0.172 | 0.0889 |
| 41 | 0.1064 | 0.1053 | 0.0756 | 0.0787 |
| 42 | 0.2312 | 0.1536 | 0.1476 | 0.1695 |
| 43 | 0.1968 | 0.1431 | 0.1153 | 0.0687 |
| 44 | 0.2156 | 0.1262 | 0.1392 | 0.1262 |
| 45 | 0.1901 | 0.2417 | 0.2325 | 0.1188 |
| 46 | 0.0754 | 0.1859 | 0.1185 | 0.0688 |
| 47 | 0.3168 | 0.1181 | 0.0925 | 0.0857 |
| 48 | 0.3719 | 0.2675 | 0.1683 | 0.1067 |
| 49 | 0.5927 | 0.3603 | 0.1933 | 0.1587 |
| 50 | 0.1881 | 0.1211 | 0.1893 | 0.0983 |
| 51 | 0.4821 | 0.2066 | 0.1677 | 0.1425 |
| 52 | 0.3927 | 0.1475 | 0.2127 | 0.1249 |
| 53 | 0.1465 | 0.104 | 0.1183 | 0.0668 |
| 54 | 0.152 | 0.1261 | 0.1092 | 0.0757 |
| 55 | 0.1692 | 0.1188 | 0.1019 | 0.0794 |
| 56 | 0.1371 | 0.1131 | 0.0971 | 0.0687 |
| 57 | 0.4915 | 0.3714 | 0.1611 | 0.0752 |
| 58 | 0.2028 | 0.1466 | 0.1413 | 0.096 |
| 59 | 0.416 | 0.2022 | 0.1399 | 0.0865 |
| 60 | 0.473 | 0.2125 | 0.1351 | 0.1911 |
| 61 | 0.1363 | 0.1828 | 0.1877 | 0.0965 |
| 62 | 0.5527 | 0.2665 | 0.1378 | 0.1083 |
| 63 | 0.5297 | 0.2944 | 0.2183 | 0.1302 |
| 64 | 0.1193 | 0.0886 | 0.0745 | 0.0907 |
| 65 | 0.1418 | 0.1794 | 0.1363 | 0.0848 |
| 66 | 0.1748 | 0.2172 | 0.1377 | 0.0811 |
| 67 | 0.1751 | 0.1407 | 0.0865 | 0.0945 |
| 68 | 0.296 | 0.1623 | 0.108 | 0.2639 |
| 69 | 0.1363 | 0.1828 | 0.1877 | 0.0965 |
| 70 | 0.1704 | 0.2179 | 0.2023 | 0.1288 |
| 71 | 0.1582 | 0.1908 | 0.1565 | 0.106 |
| 72 | 0.1962 | 0.2438 | 0.1668 | 0.1145 |
| 73 | 0.1206 | 0.1019 | 0.0756 | 0.0789 |
| 74 | 0.234 | 0.2138 | 0.1135 | 0.0939 |
| 75 | 0.1689 | 0.1223 | 0.1351 | 0.081 |
| Fungal Strains | 0 | 0.15 | 0.25 | 0.35 |
| 1F | 0.12 | 0.3344 | 0.281 | 0.2756 |
| 2F | 0.1676 | 0.392 | 0.2532 | 0.2488 |
| 3F | 0.169 | 0.212 | 0.3914 | 0.533 |
| 4F | 0.1032 | 0.3118 | 0.2699 | 0.192 |
| 5F | 0.1067 | 0.2748 | 0.1753 | 0.1612 |
| 6F | 0.3465 | 0.389 | 0.414 | 0.6342 |
| 7F | 0.3326 | 0.5194 | 0.5825 | 0.6824 |
| 8F | 0.1464 | 0.197 | 0.2146 | 0.4066 |
| 9F | 0.113 | 0.3822 | 0.4637 | 0.5491 |
| 10F | 0.2715 | 0.4058 | 0.3926 | 0.3303 |
| 11F | 0.9847 | 1.0041 | 1.171 | 1.1775 |
| 12F | 0.2509 | 0.3793 | 0.427 | 0.667 |
| 13F | 0.0725 | 0.1684 | 0.19 | 0.254 |
| 14F | 0.5349 | 0.587 | 0.568 | 0.5497 |
| 15F | 0.2658 | 0.4013 | 0.5154 | 0.6324 |
| 16F | 0.6756 | 0.969 | 0.9719 | 1.1684 |
| 17F | 0.1319 | 0.4595 | 0.5731 | 0.6546 |
| 18F | 0.5291 | 0.7085 | 0.7283 | 0.8424 |
| 19F | 0.0518 | 0.1103 | 0.1367 | 0.1643 |
| 20F | 0.3196 | 0.4908 | 0.6254 | 0.969 |
| 21F | 0.3714 | 0.5366 | 0.5241 | 0.5109 |
| 22F | 0.996 | 0.8204 | 0.4218 | 0.3796 |
| 23F | 0.1648 | 0.256 | 0.402 | 0.548 |
| 24F | 0.2306 | 0.4588 | 0.3718 | 0.3656 |
| 25F | 0.2388 | 0.4238 | 0.5231 | 0.5739 |
| 26F | 0.2556 | 0.355 | 0.4729 | 0.546 |
| 27F | 0.21 | 0.3 | 0.41 | 0.4961 |
| 28F | 0.222 | 0.39 | 0.521 | 0.5583 |

**Table S7**. The strains selected for soil inoculation.

| Isolate code | Strain |
| --- | --- |
| bacteria |  |
| 32 | *Bacillus megaterium* |
| 39 | *Bacillus endophyticus* |
| 49 | *Bacillus arbutinivorans* |
| 68 | *Streptomyces rochei* |
| Fungi |  |
| 7 | *Aspergillus terreus* |
| 11 | *Penicillium raperi* |
| 18 | *Trichoderma ghanense* |
| 16 | *Gongronella butleri* |
| 20 | *Rhizopus stolonifer* |
